# Beyond Endoscopy: A Narrative Review of the Emerging Role of Gastroenterologists in Obesity Medicine, GLP-1 (Glucagon-Like Peptide-1) Therapy, and Metabolic Care

**DOI:** 10.7759/cureus.109979

**Published:** 2026-05-31

**Authors:** Mohammad Alali, Ahmed Kunwer Naveed, Akram Alnounou, Mohamed Alharami, Bola Nashed

**Affiliations:** 1 Internal Medicine, Henry Ford Macomb Hospital, Macomb, USA; 2 Internal Medicine, Western Michigan University Homer Stryker M.D. School of Medicine, Kalamazo, USA; 3 Internal Medicine, Henry Ford Warren Hospital, Warren, USA; 4 Gastroenterology and Hepatology, Henry Ford St. John Hospital, Detroit, USA

**Keywords:** endobariatrics, esg, gastroenterology, glp-1 receptor agonists, metabolic care, metabolic dysfunction-associated steatotic liver disease, obesity medicine

## Abstract

Obesity has emerged as one of the leading global drivers of gastrointestinal and metabolic disease, contributing substantially to the burden of metabolic dysfunction-associated steatotic liver disease (MASLD), gastroesophageal reflux disease (GERD), Barrett's esophagus, pancreatitis, colorectal neoplasia, and obesity-associated malignancies. Simultaneously, the rapid expansion of glucagon-like peptide-1 (GLP-1) receptor agonists and minimally invasive bariatric endoscopic interventions has transformed the therapeutic landscape of obesity management and increasingly positioned gastroenterologists at the intersection of obesity medicine, hepatology, therapeutic endoscopy, nutrition, and metabolic care. This narrative review evaluates the evolving role of gastroenterologists in obesity medicine and discusses the integration of pharmacologic, endoscopic, metabolic, and hepatologic approaches into modern gastrointestinal practice. A focused literature review was conducted by searching PubMed and Google Scholar, prioritizing peer-reviewed publications from 2015 to 2026, with inclusion of landmark earlier studies where relevant. Articles were selected based on clinical relevance, methodological quality, and applicability to the evolving role of gastroenterologists in metabolic care. The role of gastroenterologists is expanding beyond traditional luminal and procedural practice into longitudinal metabolic disease management. The emergence of GLP-1 receptor agonists, dual incretin therapies, and endobariatric procedures has accelerated GI involvement in obesity treatment and preventive metabolic care. In parallel, the increasing prevalence of MASLD and obesity-related gastrointestinal disorders has reinforced the importance of comprehensive obesity management strategies involving gastroenterologists within multidisciplinary care teams. Integration of obesity medicine into gastroenterology training programs and metabolic clinics may further redefine the future scope of GI practice. Modern gastroenterology is undergoing a transition toward obesity-focused and metabolic care. As pharmacologic and endoscopic obesity therapies continue to evolve, gastroenterologists are uniquely positioned to play a central role in the multidisciplinary management of obesity-associated gastrointestinal disease.

## Introduction and background

Obesity represents one of the most significant public health challenges of the 21st century and is increasingly recognized as a major contributor to gastrointestinal (GI) and metabolic disease. According to the World Health Organization (WHO), global obesity rates have nearly tripled over recent decades, now affecting hundreds of millions of adults worldwide [1]. Parallel to this epidemic has been a dramatic rise in metabolic dysfunction-associated steatotic liver disease (MASLD), gastroesophageal reflux disease (GERD), Barrett's esophagus, obesity-associated GI malignancies, gallstone disease, and metabolic inflammatory conditions [2-4].

Historically, obesity management was largely directed by primary care physicians, endocrinologists, bariatric surgeons, and multidisciplinary weight-management programs, while gastroenterologists maintained a predominantly luminal and procedural focus. However, advances in pharmacologic obesity therapy and bariatric endoscopy are increasingly reshaping the role of gastroenterologists within modern metabolic care.

The emergence of glucagon-like peptide-1 (GLP-1) receptor agonists and dual incretin therapies has fundamentally transformed obesity medicine. Agents such as semaglutide and tirzepatide have demonstrated unprecedented efficacy in weight reduction and metabolic improvement, with clinical trials reporting weight loss outcomes approaching those traditionally associated with bariatric surgery [5,6]. Simultaneously, the rapid growth of bariatric endoscopy, including endoscopic sleeve gastroplasty (ESG), intragastric balloon therapy, and revision endobariatric procedures, has expanded minimally invasive obesity treatment options [7].

In parallel, obesity has become central to hepatology practice due to the rising burden of MASLD and metabolic dysfunction-associated steatohepatitis (MASH), which now represent leading causes of chronic liver disease and liver transplantation in many countries [8]. Consequently, gastroenterologists and hepatologists are increasingly involved in longitudinal obesity management, nutritional counseling, metabolic disease treatment, and multidisciplinary obesity care.

**Figure 1:**
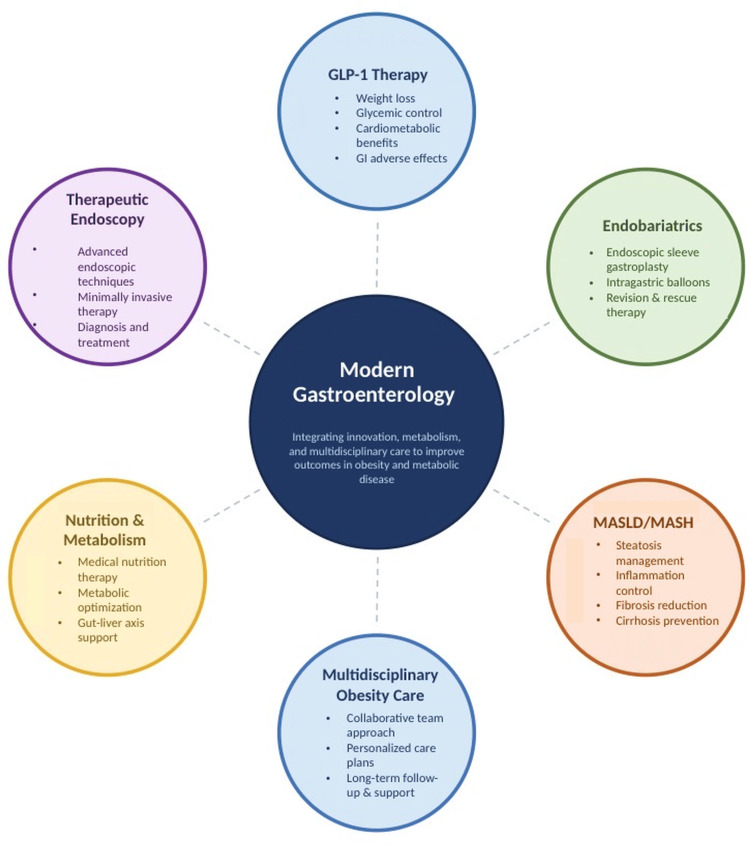
The convergence of obesity medicine, endobariatrics, and metabolic hepatology in modern gastroenterology Conceptual illustration demonstrating the expanding integration of glucagon-like peptide-1 (GLP-1)-based obesity pharmacotherapy, therapeutic endoscopy, metabolic hepatology, nutrition and metabolism, and multidisciplinary obesity care within modern gastroenterology practice. The figure highlights the evolving role of gastroenterologists in comprehensive metabolic disease management and obesity-associated gastrointestinal care. Figure created by authors using Microsoft PowerPoint (Microsoft Corporation, Redmond, Washington, United States).

The purpose of this narrative review is to address a specific and timely clinical question: how should the role of gastroenterologists be redefined in light of the convergence of GLP-1 pharmacotherapy, endobariatrics, metabolic hepatology, and evolving training paradigms? While prior reviews have addressed individual components of this transformation, this review aims to synthesize these domains into a unified framework for modern metabolic GI practice and to provide practical guidance for the integration of obesity medicine into gastroenterology. The evolving convergence of obesity medicine, endobariatrics, metabolic hepatology, and multidisciplinary metabolic care is illustrated in Figure 1.

Search strategy for review

This narrative review was conducted through a focused literature search of PubMed and Google Scholar. Search terms included: "GLP-1 receptor agonist obesity," "semaglutide weight loss," "tirzepatide obesity," "endoscopic sleeve gastroplasty," "endobariatrics," "MASLD treatment," "MASH pharmacotherapy," "gastroenterology obesity medicine," "bariatric endoscopy training," and "metabolic hepatology." Publications from January 2015 through April 2026 were prioritized, with inclusion of landmark earlier studies where clinically significant. Articles were selected based on peer-review status, clinical relevance, study design quality, and direct applicability to the evolving role of gastroenterologists in obesity and metabolic care. Case reports and non-English-language publications were excluded. Given the narrative design of this review, formal meta-analysis and quantitative synthesis were not performed.

## Review

Obesity as a GI and metabolic disease

Obesity contributes significantly to GI morbidity and mortality and is increasingly recognized as a central driver of metabolic GI disease. The relationship between obesity and GI pathology extends beyond excess adiposity alone and involves complex metabolic, inflammatory, hormonal, and microbiome-mediated pathways.

Among obesity-associated GI diseases, MASLD has emerged as one of the most clinically significant conditions. MASLD affects approximately one-quarter of the global population and has become the most common cause of chronic liver disease worldwide [8,9]. Progressive disease may lead to MASH, advanced fibrosis, cirrhosis, hepatocellular carcinoma, and liver transplantation. The rising prevalence of obesity and insulin resistance has directly contributed to the growing burden of MASLD-related complications.

Obesity also contributes significantly to GERD and Barrett's esophagus through increased intra-abdominal pressure, altered lower esophageal sphincter physiology, delayed gastric emptying, and systemic inflammation [10]. Furthermore, obesity is associated with an increased risk of colorectal neoplasia, likely mediated through insulin resistance, adipokine dysregulation, chronic inflammation, and altered gut microbiota [11].

The gut-brain axis and intestinal microbiome have emerged as additional areas linking obesity with GI disease. Dysbiosis and reduced microbial diversity may contribute to obesity-associated inflammation, metabolic dysfunction, intestinal permeability, and altered satiety signaling [12]. These observations further reinforce the expanding overlap between gastroenterology, nutrition, metabolism, and obesity medicine.

Consequently, obesity can no longer be viewed solely as an endocrine or cardiovascular condition. Rather, it increasingly represents a core GI and metabolic disease requiring multidisciplinary management strategies involving gastroenterologists, hepatologists, bariatric specialists, nutritionists, endocrinologists, and obesity medicine physicians.

The GLP-1 era and its implications for gastroenterology

The introduction of GLP-1 receptor agonists has dramatically transformed obesity medicine and metabolic disease management. Initially developed for type 2 diabetes mellitus, GLP-1-based therapies have since demonstrated substantial efficacy in weight reduction, glycemic control, cardiovascular risk reduction, and broader metabolic improvement [5,6].

In the STEP (Semaglutide Treatment Effect in People with obesity)-1 trial, once-weekly subcutaneous semaglutide produced mean total body weight reductions of approximately 14.9% over 68 weeks in adults with overweight or obesity without diabetes [5]. Tirzepatide, a dual gastric inhibitory polypeptide (GIP)/GLP-1 receptor agonist, demonstrated even greater weight reduction in the SURMOUNT-1 trial, with participants achieving mean reductions of up to 20.9% at the highest dose [6]. Notably, the recently published SURMOUNT-5 trial, the first head-to-head randomized comparison of tirzepatide versus semaglutide, demonstrated that tirzepatide produced significantly greater weight loss than semaglutide at 72 weeks, with a mean difference of approximately 10.1 percentage points in total body weight reduction, further establishing the superiority of dual incretin therapy for weight management in patients with obesity [13]. These results have substantially altered the therapeutic landscape of obesity medicine and generated unprecedented clinical and public interest in pharmacologic obesity therapy.

Beyond weight reduction, GLP-1 receptor agonists have demonstrated significant cardiometabolic benefits. The SELECT (Semaglutide Effects on Cardiovascular Outcomes in People With Overweight or Obesity) trial, a large-scale randomized controlled trial enrolling over 17,000 adults with obesity and established cardiovascular disease but without diabetes, demonstrated that once-weekly semaglutide reduced the risk of major adverse cardiovascular events by 20% compared with placebo, independent of the degree of weight loss achieved [14]. These findings have reinforced the role of GLP-1 receptor agonists as disease-modifying metabolic agents with benefits extending well beyond adiposity reduction alone.

The rapid expansion of GLP-1 therapy carries important implications for gastroenterologists. GI adverse effects, including nausea, vomiting, constipation, diarrhea, bloating, delayed gastric emptying, and abdominal pain, represent the most commonly reported side effects associated with GLP-1 receptor agonists [15]. As a result, gastroenterologists are increasingly involved in the evaluation and management of medication-related GI symptoms. Growing concerns regarding GLP-1-associated gastroparesis, bowel obstruction, nutritional complications, aspiration risk during endoscopy, and peri-procedural management have further expanded GI involvement in obesity pharmacotherapy [16]. Recent multisociety peri-procedural guidance regarding GLP-1 receptor agonist management before sedation and endoscopy further highlights the procedural implications of these agents for gastroenterology practice [17].

In hepatology, GLP-1 receptor agonists have demonstrated particularly promising effects on obesity-associated liver disease. The LEAN trial established that liraglutide significantly improved histologic features of non-alcoholic steatohepatitis (NASH) compared with placebo, including higher rates of NASH resolution and lower rates of fibrosis progression [18]. More recently, the phase 3 ESSENCE (Effect of Semaglutide in Subjects with Non-cirrhotic Environmental/Nonalcoholic Steatohepatitis) trial demonstrated that once-weekly semaglutide produced significantly greater rates of MASH resolution without worsening of fibrosis compared with placebo, representing a landmark advance in the pharmacologic treatment of MASH and establishing GLP-1 receptor agonists as a cornerstone of emerging MASH-directed therapy [19]. Furthermore, the FLOW (Evaluate Renal Function with Semaglutide Once Weekly) trial demonstrated that semaglutide significantly reduced the risk of kidney disease progression and cardiovascular death in patients with type 2 diabetes and chronic kidney disease, highlighting the broad organ-protective potential of GLP-1 receptor agonists in patients with obesity-related metabolic complications [20]. These findings collectively suggest that GLP-1 receptor agonists will play an increasingly central role in metabolic hepatology and the comprehensive management of obesity-associated organ damage.

Beyond pharmacotherapy itself, the emergence of GLP-1 therapy reflects a broader shift toward longitudinal obesity management and preventive metabolic care. Gastroenterologists are increasingly participating in patient selection, nutritional counseling, metabolic risk stratification, endoscopic evaluation, and multidisciplinary obesity treatment pathways.

Evolution of endobariatrics

Endobariatrics has emerged as one of the fastest-growing areas within therapeutic gastroenterology. Endoscopic bariatric therapies offer minimally invasive alternatives to traditional bariatric surgery and occupy an important therapeutic space between lifestyle intervention and surgical treatment for patients with obesity.

Among these interventions, endoscopic sleeve gastroplasty (ESG) has become one of the most widely adopted endobariatric procedures. ESG utilizes full-thickness endoscopic suturing to reduce gastric volume without surgical resection and has demonstrated clinically meaningful reductions in total body weight and metabolic risk factors [21]. Compared with surgical approaches, ESG may offer lower procedural morbidity, shorter recovery times, and improved accessibility for selected patients who are not surgical candidates or prefer less invasive options. In a prospective study, ESG resulted in approximately 15-20% total body weight loss at one year alongside improvements in metabolic parameters including blood pressure, lipid profiles, and glycemic control [21].

Additional endobariatric interventions include intragastric balloons, aspiration therapy, endoscopic revision procedures following bariatric surgery, and investigational metabolic endoscopic therapies [7,22]. These procedures continue to evolve alongside increasing demand for minimally invasive obesity treatment options.

The relationship between pharmacologic obesity therapy and endobariatrics remains an area of active investigation. While some authors have raised questions about whether highly effective GLP-1 receptor agonists may reduce demand for bariatric procedures over time, emerging evidence suggests that pharmacologic and endoscopic therapies may function as complementary rather than competing modalities. A comparative effectiveness analysis by Haseeb et al. found that semaglutide and ESG produced broadly similar weight loss outcomes at one year, suggesting a potential role for individualized treatment selection based on patient preference, comorbidities, and risk profile [23]. Emerging interest has focused on combination approaches utilizing GLP-1 receptor agonists alongside ESG and other endobariatric procedures, with early data suggesting potential synergistic metabolic benefits; however, long-term comparative outcomes, durability, and optimal treatment sequencing remain areas of ongoing investigation.

In addition, procedural availability, insurance coverage limitations, and variability in specialized endobariatric training continue to represent important barriers to broader implementation. Importantly, endobariatrics represents more than procedural innovation alone. The growth of bariatric endoscopy reflects a broader transformation within gastroenterology itself, emphasizing longitudinal obesity management, multidisciplinary metabolic care, and preventive intervention rather than isolated procedural treatment.

**Figure 2:**
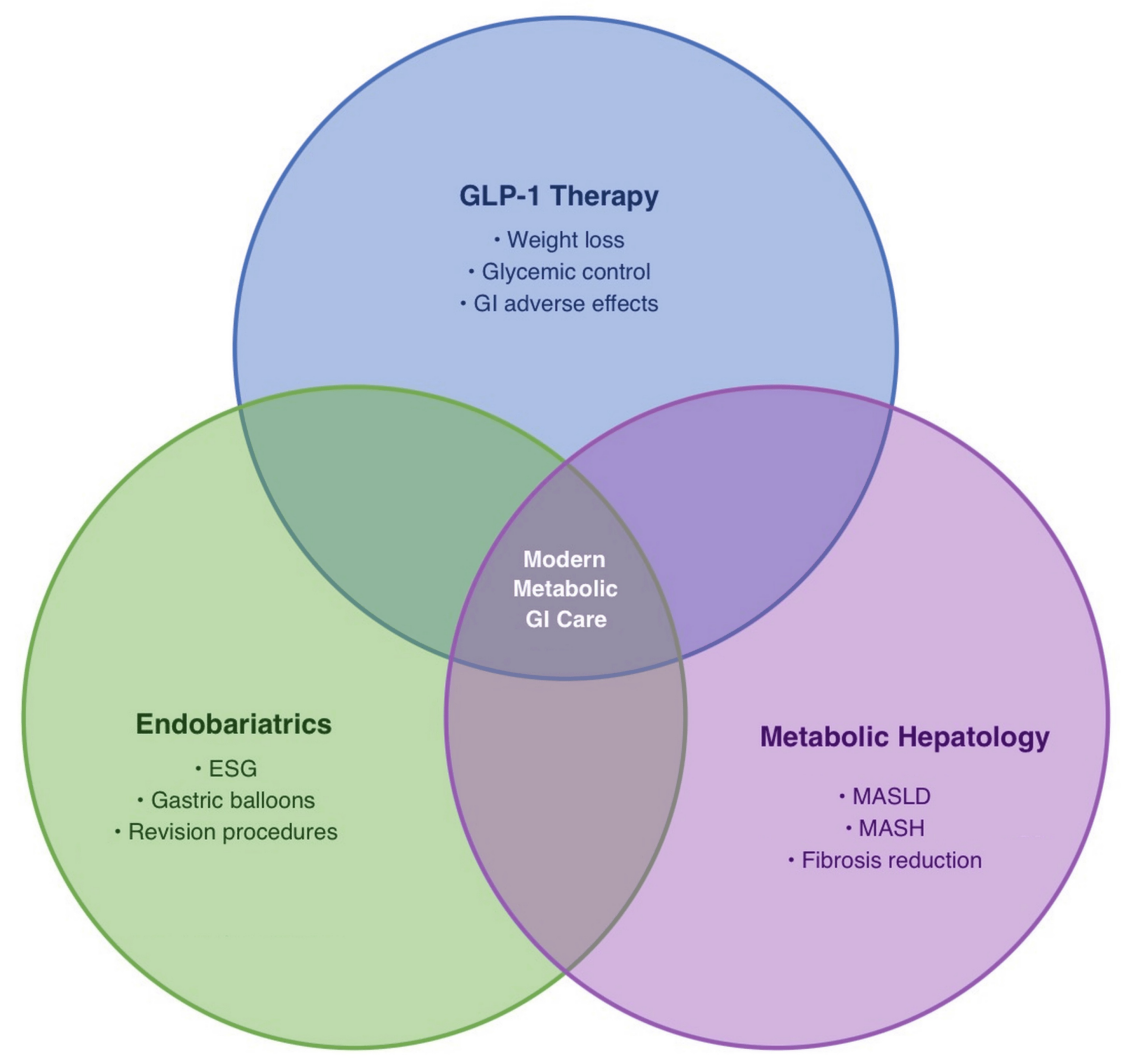
Intersection of GLP-1 therapy, endobariatrics, and metabolic hepatology. Conceptual Venn diagram demonstrating the overlap between GLP-1-based obesity pharmacotherapy, bariatric endoscopic interventions, and metabolic hepatology within modern gastroenterology practice. The central intersection highlights the emergence of integrated metabolic GI care, emphasizing multidisciplinary obesity management, metabolic disease treatment, and comprehensive care of obesity-associated gastrointestinal and liver disease. Figure created by authors using Microsoft PowerPoint (Microsoft Corporation, Redmond, Washington, United States).

Increasing overlap between pharmacologic obesity therapy, bariatric endoscopy, and metabolic hepatology is contributing to the emergence of integrated metabolic GI care models, as illustrated in Figure 2.

MASLD and metabolic hepatology

The increasing prevalence of MASLD has significantly reshaped hepatology practice. Closely linked to obesity, insulin resistance, type 2 diabetes mellitus, and metabolic syndrome, MASLD now represents one of the leading causes of chronic liver disease worldwide [8,9].

Weight reduction remains among the most effective interventions for improving hepatic steatosis, inflammation, and fibrosis progression. Studies have demonstrated that weight loss of 7-10% of total body weight is associated with significant histologic improvement in NASH, while losses exceeding 10% may produce fibrosis regression [24]. Consequently, therapies capable of producing sustained, meaningful weight loss have become central to metabolic hepatology practice.

GLP-1 receptor agonists have demonstrated promising effects on hepatic steatosis and metabolic parameters in patients with MASLD and MASH, with mechanisms including weight reduction, improved insulin sensitivity, decreased hepatic inflammation, and modulation of hepatic lipid metabolism [18]. Similarly, bariatric surgery and endobariatric procedures may provide additional hepatic benefits in selected patients with obesity-associated liver disease [25]. The concept of "metabolic hepatology" reflects the growing intersection between obesity medicine, hepatology, endocrinology, and preventive care.

Recent advances in MASH-directed pharmacotherapy and noninvasive fibrosis assessment may further accelerate the evolution of metabolic hepatology. The approval of resmetirom, a liver-directed thyroid hormone receptor-beta agonist, for the treatment of MASH with liver fibrosis (stages F2-F3) in 2024 represents a landmark advance in targeted MASH pharmacotherapy [26]. Increasing utilization of fibrosis risk stratification tools such as FibroScan, FIB-4, and enhanced liver fibrosis (ELF) testing has expanded opportunities for early identification and longitudinal monitoring of obesity-associated liver disease. As the prevalence of obesity and MASLD continues to rise, gastroenterologists and hepatologists are likely to play an increasingly central role in multidisciplinary obesity management programs.

Why gastroenterologists are uniquely positioned in obesity medicine

Gastroenterologists occupy a unique position within obesity medicine due to their expertise in therapeutic endoscopy, hepatology, nutrition, GI physiology, and longitudinal digestive care [27].

Unlike many specialties involved in obesity management, gastroenterologists are able to integrate procedural, metabolic, nutritional, and hepatologic aspects of care within a single specialty framework. Obesity-associated GI disease frequently involves overlapping pathology, including GERD, MASLD, altered gut motility, nutritional deficiencies, bariatric anatomy, and GI complications of pharmacologic therapy.

The increasing convergence of obesity medicine, therapeutic endoscopy, hepatology, and metabolic disease management has positioned gastroenterologists to function as central coordinators within multidisciplinary metabolic care models. This evolving role extends beyond procedural intervention and increasingly incorporates longitudinal risk stratification, preventive care, and chronic metabolic disease management.

**Figure 3:**
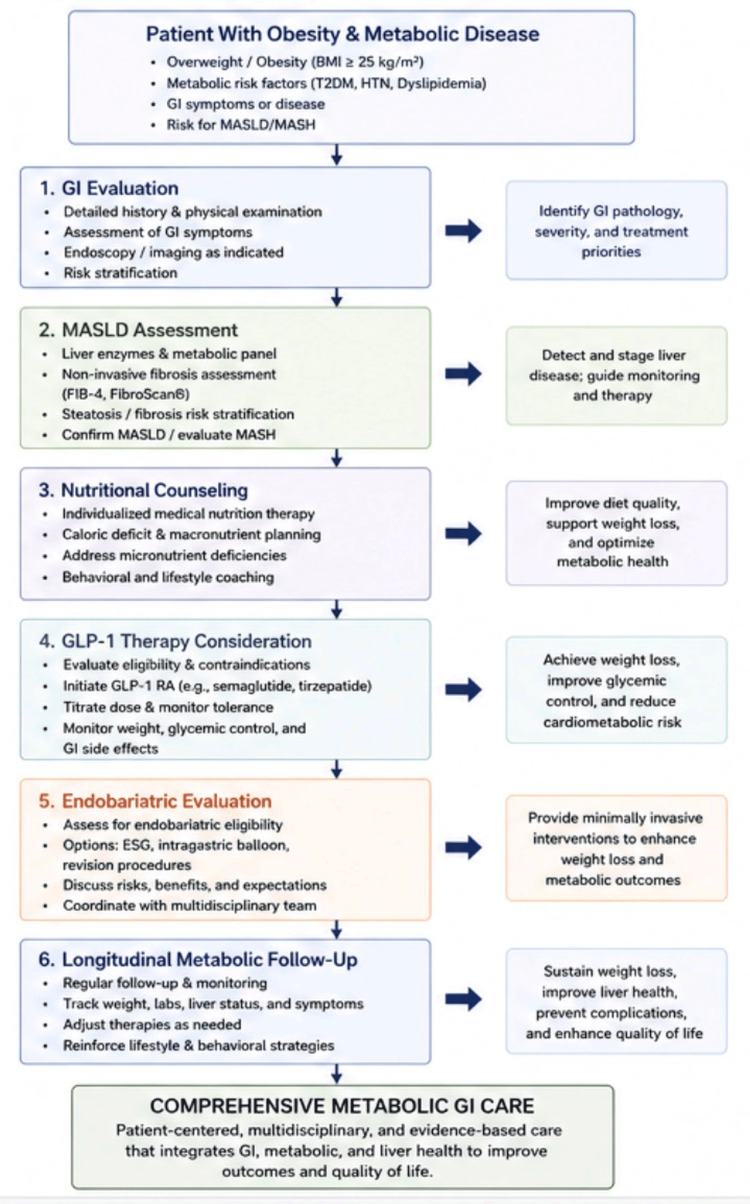
Integrated metabolic GI care pathway for patients with obesity-associated gastrointestinal disease. Proposed multidisciplinary clinical pathway illustrating the comprehensive evaluation and management of patients with obesity-associated gastrointestinal and metabolic disease. The pathway integrates gastrointestinal assessment, MASLD evaluation, nutritional counseling, GLP-1-based pharmacotherapy, endobariatric intervention, and longitudinal metabolic follow-up within a patient-centered model of modern metabolic GI care. MASLD: metabolic dysfunction-associated steatotic liver disease; GLP-1: glucagon-like peptide-1; T2DM: type 2 diabetes mellitus; HTN: hypertension; MASH: metabolic dysfunction-associated steatohepatitis; RA: receptor agonist; ESG: endoscopic sleeve gastroplasty; FIB-4: Fibrosis-4 Index Figure created by authors using Microsoft PowerPoint (Microsoft Corporation, Redmond, Washington, United States).

Furthermore, gastroenterologists possess expertise in minimally invasive intervention through bariatric endoscopy and are increasingly participating in multidisciplinary obesity clinics alongside endocrinologists, bariatric surgeons, psychologists, nutritionists, and obesity medicine specialists [28]. A proposed integrated metabolic GI care pathway for patients with obesity-associated GI disease is illustrated in Figure 3.

The expanding role of gastroenterologists in obesity medicine also reflects broader changes in healthcare emphasizing chronic disease management, preventive care, and metabolic health optimization. Rather than functioning solely as procedural consultants, gastroenterologists are increasingly involved in longitudinal obesity care and multidisciplinary metabolic management. This evolving role may ultimately reshape the future identity of gastroenterology itself, integrating obesity medicine, metabolic disease, hepatology, therapeutic endoscopy, and preventive care into a more comprehensive model of GI practice.

Challenges and controversies in metabolic GI care

Despite the rapid expansion of obesity-focused GI care, several important clinical and healthcare-system challenges remain unresolved. Although GLP-1 receptor agonists and endobariatric therapies have demonstrated substantial efficacy in weight reduction and metabolic improvement, widespread implementation of these interventions continues to face barriers related to cost, insurance coverage, long-term adherence, and healthcare accessibility. In many healthcare systems, obesity pharmacotherapy remains inconsistently reimbursed despite growing recognition of obesity as a chronic metabolic disease requiring longitudinal treatment [4,6].

Long-term durability of obesity therapy also remains an important consideration. The STEP 4 trial demonstrated that discontinuation of semaglutide was associated with significant weight regain, underscoring the chronic relapsing nature of obesity and the need for sustained pharmacologic management strategies [29]. In addition, GI adverse effects, medication shortages, and variability in patient response may complicate long-term treatment strategies.

Similarly, while endobariatric procedures such as ESG have demonstrated promising outcomes, long-term comparative data regarding durability, cost-effectiveness, and optimal integration with pharmacologic therapy remain limited [23]. Ongoing uncertainty also surrounds the future role of combination metabolic therapy utilizing GLP-1 receptor agonists alongside bariatric endoscopic interventions. As obesity medicine continues to expand within gastroenterology, future progress will depend on the development of evidence-based multidisciplinary care models capable of integrating pharmacologic, endoscopic, nutritional, and metabolic approaches into sustainable long-term patient management.

Implications for gastroenterology training and workforce development

The rapid expansion of obesity medicine raises important questions regarding the future of gastroenterology training. Traditional GI fellowship curricula have historically focused on luminal disorders, inflammatory bowel disease, hepatology, and diagnostic or therapeutic endoscopy, with limited formal training in obesity medicine and metabolic disease management [30].

However, the emergence of GLP-1-based therapy, endobariatrics, and metabolic hepatology suggests a growing need for obesity-focused education within gastroenterology fellowship programs. Future training paradigms may increasingly incorporate obesity pharmacotherapy, metabolic disease management, bariatric endoscopy, nutritional medicine, and multidisciplinary obesity care.

Specialized training pathways in bariatric endoscopy and obesity medicine are already emerging at selected academic centers [30]. As demand for minimally invasive obesity therapies continues to increase, expertise in metabolic GI care may become an increasingly valuable component of modern gastroenterology practice. In parallel, growing interest in obesity medicine certification and advanced metabolic endoscopy training may further expand the role of gastroenterologists within multidisciplinary obesity care models.

Additionally, future workforce demands may require closer collaboration between gastroenterologists, endocrinologists, bariatric surgeons, and obesity medicine physicians. The growing overlap between hepatology, obesity medicine, and metabolic disease management may further accelerate the development of multidisciplinary metabolic GI programs [31]. Ultimately, the continued integration of obesity medicine into gastroenterology training may help redefine the future identity of the specialty toward a more comprehensive model of metabolic GI care.

Future directions

The future of obesity medicine within gastroenterology will likely involve increasing integration of pharmacologic, endoscopic, nutritional, and digital health approaches. Combination strategies utilizing GLP-1 receptor agonists alongside endobariatric procedures may become increasingly common and warrant further prospective investigation, particularly with regard to long-term weight maintenance, metabolic outcomes, and cost-effectiveness [23]. As obesity management continues to evolve, individualized treatment pathways integrating pharmacotherapy, bariatric endoscopy, nutritional intervention, and metabolic risk stratification may become central components of modern GI practice.

Advances in artificial intelligence, precision medicine, microbiome science, and metabolic phenotyping may further personalize obesity treatment strategies and expand the role of gastroenterologists in metabolic disease management [12]. In parallel, the continued development of targeted therapies for MASLD and MASH, including resmetirom and investigational agents targeting hepatic fibrosis pathways, may further strengthen the integration of obesity medicine, hepatology, and therapeutic endoscopy within multidisciplinary metabolic care models [26].

Ongoing increases in obesity prevalence and MASLD-related disease burden will likely continue to strengthen the relationship between obesity medicine, hepatology, and therapeutic endoscopy. Future research should focus on long-term outcomes of combined pharmacologic and endoscopic obesity therapies, optimization of multidisciplinary metabolic care models, and development of evidence-based obesity medicine curricula within gastroenterology training programs.

Limitations

As a narrative review, this manuscript is inherently subject to selection bias and does not utilize systematic review methodology or quantitative meta-analysis. The literature search was focused rather than exhaustive, and the selection of studies reflects the authors' clinical judgment regarding relevance and quality. Additionally, many aspects of obesity medicine and endobariatrics remain rapidly evolving, and long-term comparative outcomes data regarding combined pharmacologic and endoscopic obesity therapy remain limited. Rapid advances in obesity pharmacotherapy, metabolic hepatology, and bariatric endoscopy may continue to reshape clinical practice and therapeutic paradigms as additional long-term evidence emerges. Readers should interpret the clinical guidance offered in this review within the context of these limitations and in conjunction with available society guidelines.

## Conclusions

Gastroenterology is undergoing a significant transformation driven by the expanding burden of obesity-associated GI disease and the rapid evolution of obesity therapeutics. The emergence of GLP-1 receptor agonists, minimally invasive bariatric endoscopy, and metabolic hepatology has broadened the role of gastroenterologists beyond traditional procedural and luminal care. Modern gastroenterologists are increasingly positioned at the intersection of obesity medicine, metabolic disease, hepatology, nutrition, and therapeutic endoscopy. As obesity-focused therapies continue to evolve, the integration of metabolic care into gastroenterology practice may fundamentally reshape the future identity of the specialty.
